# Effect of Particle Size on Current-Carrying Friction and Wear Properties of Copper-Graphite Composites by Spark Plasma Sintering

**DOI:** 10.3390/ma12172825

**Published:** 2019-09-02

**Authors:** Zhenghai Yang, Yuexin Ge, Xu Zhang, Bao Shangguan, Yongzhen Zhang, Yao Wang

**Affiliations:** 1 School of Materials Science and Engineering, Henan University of Science and Technology, National Laboratory of High-end Bearing Tribology Technology and Application, Luoyang 471023, China (Y.G.) (X.Z.) (B.S.) (Y.Z.) (Y.W.); 2 State Key Laboratory of Solid Lubrication, Lanzhou Institute of Chemical Physics, Chinese Academy of Sciences, Lanzhou 730000, China

**Keywords:** particle size, copper-graphite composites, current-carrying friction, wear

## Abstract

Copper-graphite composites were prepared by spark plasma sintering (SPS) with copper powder and copper-coated graphite powder. The effect of particle size of raw material powder on the current-carrying friction properties of copper-graphite composites was studied. The results show that the friction coefficient of the composites decreased with the decrease of the particle size of copper-coated graphite powder, the friction coefficient of the composites increased with the decrease of the particle size of the copper powder, the wear rate of the composites increased with the decrease of the particle size of the copper-coated graphite powder, and the wear rate of the composites increased significantly with the decrease of the particle size of the copper-coated graphite powder. The current carrying properties of composites with different particle size ratios and QCr0.5 pairs are good and fluctuate little. The current-carrying friction properties of 150 μm copper powder and 75 μm copper-coated graphite powder were found to be the best. The wear surface could be divided into mechanical wear area and arc erosion area. The main area of arc erosion was less than 15% of the total area, and it was mainly distributed in the friction outlet area. The main forms of mechanical wear included furrow, rolling deformation, cold welding, and tearing, among other forms. Graphite film was formed on the surface. The surface quality of the composite prepared by 150 μm copper powder and 75 μm copper-coated graphite powder was the best, the Sa was 3.22 μm, rolling deformation was the most adequate, no large tear pit and furrow appeared, and the carbon content on the worn surface was much higher than that in the composite. The behavior of arc erosion was mainly melting and splashing, and the particle size of the original powder had little effect on it.

## 1. Introduction

Copper-carbon (graphite, carbon fiber, carbon nanotubes, graphene and other sp2 hybrid carbon materials) composites are widely used as current carrying friction materials [1], which are applied in the fields of electric brushes, high and low pressure contacts, and skateboards, among other items. Friction and wear properties [2] and current carrying properties [3] are the most important properties of copper-carbon composites for current-carrying friction. The copper matrix forms a three-dimensional network structure in the composites [4], which meets the requirements of electrical conductivity and mechanical properties in the service process of the materials. Carbon materials mainly play a lubrication role, make the friction pair run smoothly [5], and improve the friction and wear properties of the contact interface [6,7]. With the development of science and technology, the working conditions of materials in service are more and more harsh, even within extreme working conditions, and the requirements for material properties are higher and higher.

Powder metallurgy is a common preparation method of copper-carbon composites, with the properties of copper-carbon composites being affected by many factors. The particle size of raw material powder [8,9] affects the sintering process of the material, thus affecting the density and other parameters of the sintered material. Much research has been carried out on the influencing factors of material properties in powder metallurgy, including the sintering process [10], sintering temperature and time [11,12], composition and content of materials [13,14], and the service conditions and environment [15,16]. Hot pressing sintering [17], laser sintering [18], and other conventional powder metallurgy processes have a long sintering time and cause great change in the state of raw materials. Therefore, there are few reports on the effect of raw material particle size on the current-carrying friction properties of sintered materials.

Spark plasma sintering (SPS) is a new powder metallurgy process that has the characteristics of uniform heating, fast heating rate, low sintering temperature, short sintering time, high production efficiency, fine and uniform microstructure, and high density materials [19,20]. However, the process also maintains the natural state of raw materials, resulting in the effect of the original powder on the properties of the material being more obvious [21]. Therefore, copper-graphite composites were prepared by SPS process with different particle sizes of copper and copper-coated graphite powder, and the effect of original powder particle size on the current-carrying friction properties of sintered composites was studied.

## 2. Experimental Materials and Methods

### 2.1. Material Preparation

The test pin sample was a copper-graphite composite prepared by spark plasma sintering. The raw materials were electrolytic pure copper powder and copper-coated graphite powder with a purity of more than 99% (Beijing Xingrong Yuan Technology Co., Ltd., Beijing, China). The particle sizes were 150, 75, and 45 μm (D50). The graphite content in the copper-coated graphite powder was 50 wt%. The content of graphite in the prepared material was 7.5 wt%. The preparation process of the material is as follows: the powder was mixed on a V-type mixer for 18 h; the rotating speed was 60 rpm and was sintered in an SPS furnace in vacuum. The sintering pressure was 30 MPa, the sintering temperature was 780 °C, the temperature rise rate was 100 °C/s, and the holding time was 5 min. After sintering, it the mixture was cooled in the furnace. Finally, the sintered material was cut into pin specimens of ∅10 mm by wire cutting.

### 2.2. Performance Testing

**Figure 1:**
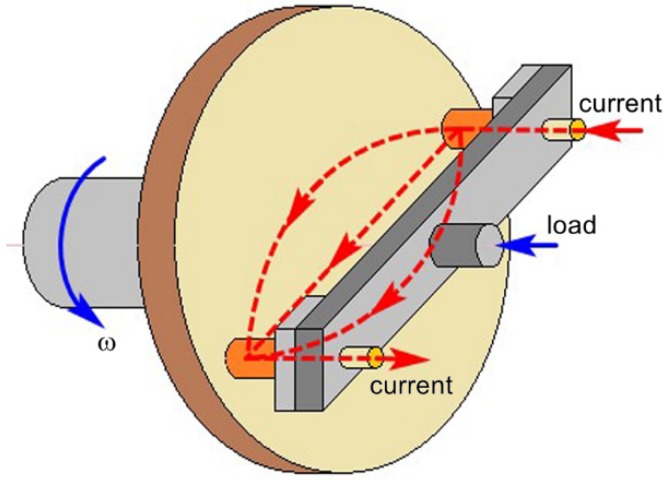
Basic schematic of the HST-100 high speed pin-on-disc tribo-tester.

The friction and wear tests were carried out on a self-made HST-100 high-speed friction test machine (see Figure 1). The friction pair was pin-disc type, with the current flowing out of one pin specimen, through the disc specimen, and back from the other pin specimen. The material of the disc specimen was QCr0.5, the positive pressure 70 N, the current 100 A, the relative sliding speed 20 m/s, and the test time 30 s. Before the test, the specimen was polished with 800# sandpaper and preground on the test machine without electricity for 10 min at the speed of 5 m/s.

The wear surface was observed by JSM-5610LV scanning electron microscope (SEM, JEDL, Tokyo, Japan) with energy-dispersive X-ray spectroscopy (EDS), the conductivity was measured by a Sigma2008B/C digital eddy current metal conductivity instrument (Shanghai, China), the density was measured by drainage method, the hardness was measured by a HV-1000 micro-hardness tester (Laizhou, China), and the surface roughness was measured by a nano Focus 3D topography instrument (Oberhausen, Germany).

In the current-carrying friction and wear test, the friction and wear properties were evaluated by the friction coefficient and the mass wear rate. The current-carrying property was evaluated by current-carrying efficiency and current-carrying stability. The current-carrying efficiency represents the ability of the friction pair to transmit the current during the service process, which is the ratio of the average value of the actual current to the given current in the service process [22]. Current-carrying stability characterizes the fluctuation of conduction current during the service of friction pair. The calculation formula is as follows:

$$\delta =\left(1-\frac{\sigma }{\bar{I_{i}}}\right)\times 100\%,$$

where δ is the current-carrying stability parameter as a percentage, and is dimensionless, with the larger the value, the higher the current-carrying stability; σ is the standard deviation for current, A; $\bar{I_{i}}$ is the average value of the actual current in the course of the test, A.

## 3. Results and Analysis

### 3.1. Microstructure, Density, Hardness and Conductivity of the Prepared Materials

**Figure 2:**
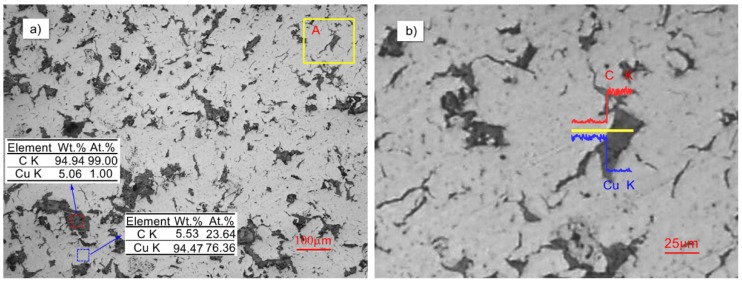
The scanning electron microscope (SEM) microstructure and EDS of Cu–C composite materials: (**a**) the SEM of copper graphite composite, (**b**) the line scan diagram of region A in (**a**).

Figure 2a shows the microstructure of copper-graphite composite prepared from 150 μm copper powder and 150 μm copper-coated graphite powder, and Figure 2b is the line scan of the interface in the A region of Figure 2a. As can be seen from the figure, the gray copper matrix formed a network structure, and the black graphite phase was uniformly distributed in the material. It can be seen from the line scan that the copper content was relatively high at first, but dropped sharply when crossing the boundary, and then was relatively stable, while the carbon content began at a low level, rose sharply when crossing the boundary, and then stabilized, with there being no overlap between the two. The results show that when the specimen was sintered by SPS, the interface of the composite was tightly bonded, and no obvious pores or cracks existed. At the same time, there was basically no coexistence zone between the two elements, which also indicated that Cu and C belong to completely incompatible elements, that the interface was mechanically combined, and that there was no reaction product [23].

**Figure 3:**
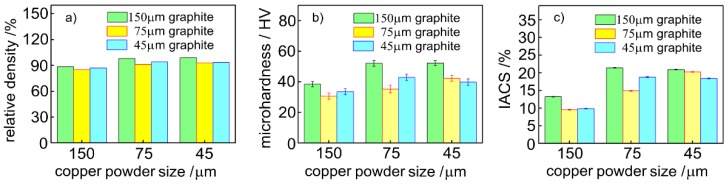
Obtained copper-graphite composite materials relative to the density (**a**), hardness (**b**), and electrical conductivity (**c**).

Figure 3 shows the density, hardness, and electrical conductivity of the copper-graphite composite with different particle size ratios. As can be seen from Figure 3a, the density of the nine materials fluctuated at about 90%, and the density increased slightly with the decrease of the particle size of the copper powder. With the decrease of the particle size of the copper-coated graphite powder, the density decreased slightly. It can be seen from Figure 3b that the hardness of the composites tended to decrease with the decrease of the particle size of the copper-coated graphite powder. The hardness of the composites increased with the decrease of the particle size of the copper powder. It can be seen from Figure 3c that as the particle size of the copper-clad graphite powder decreased, the electrical conductivity of the composite material decreased slightly; with the decrease of the particle size of the copper powder, the electrical conductivity of the composite increased.

The main factors affecting the density of materials were the number and size of voids on the interface of materials. The density of the nine kinds of materials was not significantly different, but the number of interfaces (copper/copper interface and copper/graphite interface) in the materials was significantly different, which indicated that the bonding of the nine materials was relatively dense. In the material, the copper matrix formed a three-dimensional network structure, and the copper/copper interface bonding was dense and metallurgical. Although the copper/graphite interface was dense, it was only physically bonded. As the particle size of copper-coated graphite decreased, the copper/graphite interface increased, the strength of the composite decreased, and the hardness of the composite decreased. The conductivity of the copper/copper interface was much higher than that of the copper/graphite interface. As the particle size of copper-coated graphite decreased, the conductivity of the material decreased.

### 3.2. Effect of Particle Size on Friction and Wear Properties of Materials

**Figure 4:**
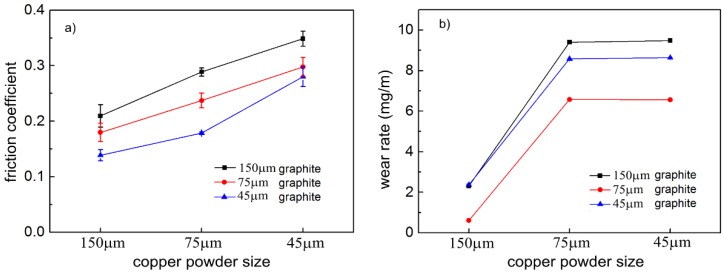
Friction and wear properties of the copper-graphite composites: (**a**) friction coefficient, (**b**) wear rate.

Figure 4 shows the friction coefficient and wear rate of copper-graphite composites with different particle size ratios. From Figure 4a, it can be seen that the friction coefficient of the composite decreased with the decrease of the particle size of the copper-coated graphite powder, and increased with the decrease of the particle size of the copper powder. It can be seen from Figure 4b that the wear rate of the composites increased with the decrease of the particle size of the copper coated graphite powder, and the wear rate of the composites increased significantly with the decrease of the particle size of the copper powder. By comparing the friction and wear properties of 150 μm copper powder with 75 μm graphite powder, the friction and wear properties of the composites prepared by 150 μm copper powder and 75 μm graphite powder were found to be the best.

### 3.3. Effect of Particle Size on Electrical Conductivity of Composite Pairs

**Figure 5:**
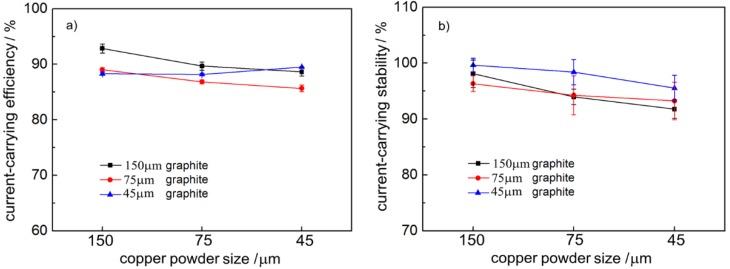
Current-carrying properties of the copper-graphite composites: (**a**) current-carrying efficiency, (**b**) current-carrying stability.

Figure 5 shows the current carrying performance data curve of the copper-graphite composite and QCr0.5 pair. The figure shows that the current-carrying efficiency of the pair fluctuated slightly between 86% and 93%, with the current carrying stability decreasing slightly with the decrease of the particle size of the copper powder, and the overall current-carrying stability being between 93% and 99%.

### 3.4. Current-Carrying Friction Behavior of Composites

**Figure 6:**
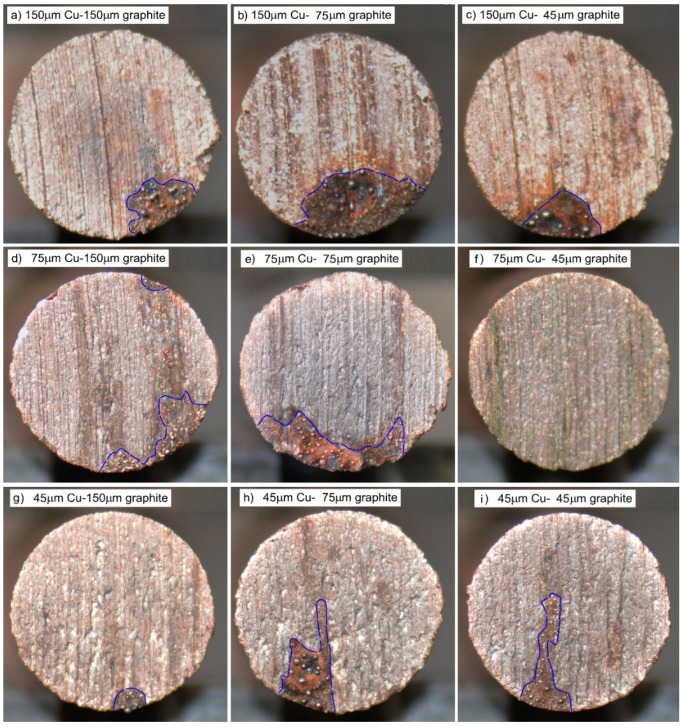
The macroscopic morphology of the friction surface: (**a**) 150 μm copper–150 μm graphite, (**b**) 150 μm copper–75 μm graphite, (**c**) 150 μm copper–45 μm graphite, (**d**) 75 μm copper–150 μm graphite, (**e**) 75 μm copper–75 μm graphite, (**f**) 75 μm copper–45 μm graphite, (**g**) 45 μm copper–150 μm graphite, (**h**) 45 μm copper–75 μm graphite, (**i**) 45 μm copper–45 μm graphite.

Figure 6 shows the macropicture of the wear surface of the copper-graphite composites prepared by different particle size raw materials. It can be seen from the diagram that the wear surface can be divided into two parts: the main area of mechanical wear and the area dominated by arc erosion, in which the area of mechanical wear was dominant. There were a few traces of arc ablation on all wear surfaces, and the maximum area of arc erosion was not more than 15% of the total area, and was mainly distributed in the area at the exit of the wear surface. With the decrease of the particle size of the original copper powder, the area of the arc ablation area tended to decrease, but fluctuated, and the relationship between the area of the main area of arc ablation and the particle size of the copper-coated graphite powder was not clear.

**Figure 7:**
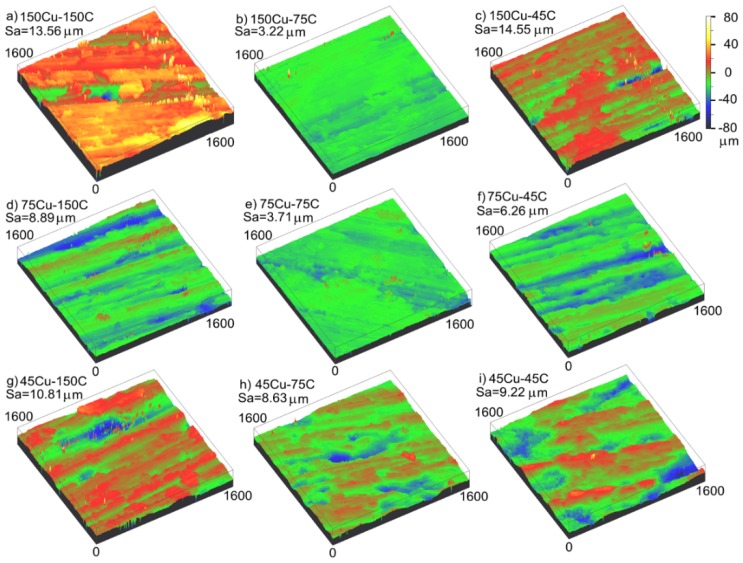
The three-dimensional morphology and surface roughness of the mechanical wear area: (**a**) 150 μm copper–150 μm graphite, (**b**) 150 μm copper–75 μm graphite, (**c**) 150 μm copper–45 μm graphite, (**d**) 75 μm copper–150 μm graphite, (**e**) 75 μm copper–75 μm graphite, (**f**) 75 μm copper–45 μm graphite, (**g**) 45 μm copper–150 μm graphite, (**h**) 45 μm copper–75 μm graphite, (**i**) 45 μm copper–45 μm graphite.

Figure 7 shows the three-dimensional morphology and surface roughness of the copper-graphite composites prepared by different particle size raw materials. Figure 7 shows that, when the particle size of the copper coated graphite powder was 150 μm and 45 μm, with the decrease of copper powder particle size, the surface roughness of the mechanical wear zone decreased at first and then increased. When the particle size of the copper-coated graphite powder was 75 μm, the surface roughness of the mechanical wear area increased as the particle size of the copper powder decreased; with the decrease of the particle size of the copper-coated graphite powder, the surface roughness of the mechanical wear area decreased at first and then increased. The surface roughness of the composites prepared by 150 μm copper powder and 75 μm copper-coated graphite powder was found to be the best, and the Sa was 3.22 μm. A few deep pits and wide and deep furrows appeared in Figure 7a,c. The surface in Figure 7b,e was smooth; at the same time, with the decrease of the copper powder particle size, the morphology of the furrows became intermittent.

Table 1 shows the main element contents in the energy spectrum analysis of the mechanical wear region corresponding to Figure 7. It can be seen from the table that there were three main elements of Cu, C, and O on the wear surface, and that the carbon content on the friction surface was much higher than that in the composite material. With the decrease of the particle size of the copper powder, the content of Cu decreased and the content of carbon increased. The particle size of the copper-coated graphite powder had little effect on the surface content.

**Figure 8:**
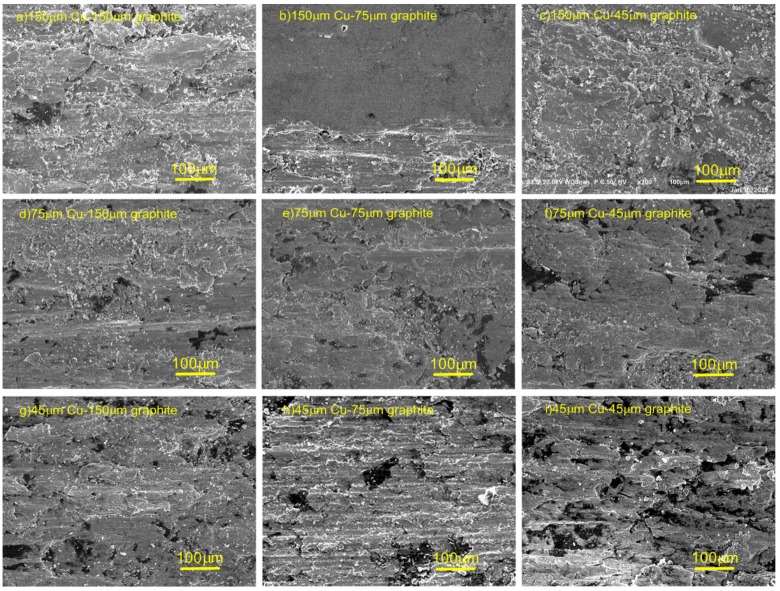
SEM photographs of the mechanical wear area of composite materials: (**a**) 150 μm copper–150 μm graphite, (**b**) 150 μm copper–75 μm graphite, (**c**) 150 μm copper–45 μm graphite, (**d**) 75 μm copper–150 μm graphite, (**e**) 75 μm copper–75 μm graphite, (**f**) 75 μm copper–45 μm graphite, (**g**) 45 μm copper–150 μm graphite, (**h**) 45 μm copper–75 μm graphite, (**i**) 45 μm copper–45 μm graphite.

Figure 8 shows the SEM photograph of the mechanical wear area of composites with different particle size ratios. As shown in Figure 8, the flake structure of copper was formed by rolling deformation. With the decrease of the particle size of the original copper powder, the size of the sheet decreased. Figure 8b shows that the deformation of flake structure was the most adequate. The furrow morphology can be seen in Figure 8, and the deformation was intermittent in Figure 8d–i, especially in Figure 8h,i. The image of cold welding tear on the wear surface is not obvious.

**Figure 9:**
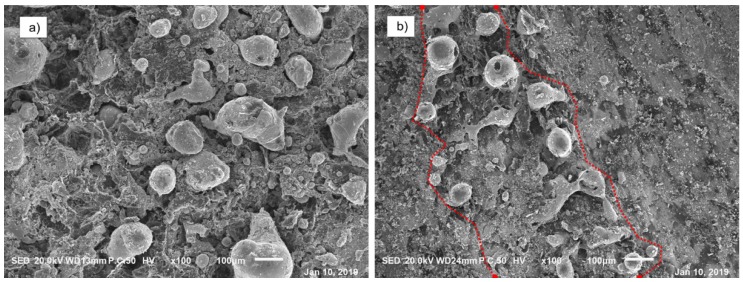
SEM photography of current-carrying wear surface of copper-graphite composites: (**a**) the picture of the large area of arc erosion, (**b**) the picture of the local area of arc erosion.

Figure 9 is a SEM photograph of the arc erosion area on the current-carrying wear surface of the copper-graphite composites. Figure 9a is a photograph of the large area of arc erosion. It can be seen from the diagram that all areas were eroded by arc, that irregular metal solidification particles of larger size appeared, and that copper particles of a smaller size and shaped similar to stars existed at the same time. Figure 9b is a photograph of the morphology of the local area eroded by the arc. The area between the two red lines in the figure is the area where the arc was seriously eroded, with large irregular metal solidified particles similar to ejected metal solidified particles, and smaller approximate spherical particles.

## 4. Discussion

In the current-carrying friction process of composite materials, two types of behaviors are mainly included—mechanical friction and wear and arc erosion [24,25]. Mechanical friction and wear behavior is the friction and wear behavior that occurs during the contact between two rough surfaces, relative sliding, and current conduction. The surface of the friction pair includes the copper micro-convex peak and the graphite micro-convex peak. There are falling graphite particles and copper particles between the two friction surfaces. There are adsorbents on the surface of these substances and oxides on the surface of the copper micro-convex peaks. Since friction and wear continue, the surface changes constantly, and graphite has the effect of inhibiting copper oxidation, thus the total amount of adsorbate and oxide on the surface is small.

When a copper micro-convex peak and copper micro-convex peak form contact and slide relatively, surface adsorbents and oxides are the first to contact. Under the action of pressure and motion, after destroying the non-conductive layer of the surface (forming conductive α spots, starting to conduct electricity until contact failure), the metal is in direct contact, and cold welding and tearing occur [26]. When the pressure and relative motion are too small to destroy the surface non-conducting layer, furrow and plastic deformation will occur. The contact between the two copper micro-convex peaks is also affected by the shape and quantity of the micro-convex peaks. Contact resistance heat reduces the strength of the copper micro-convex peak and aggravates the degree of cold welding. Tearing often occurs where the strength is low, that is, from the junction of copper powder and copper powder, so the particle size of the original copper powder affects the size of the tear product and the morphology of the friction surface. The morphology of the friction surface will further affect the shape and number of contact micro-convex peaks.

When the copper micro-convex peak is in contact with the graphite micro-convex peak and the graphite micro-convex peak is in contact with another graphite micro-convex peak, because of the layered structure of graphite, graphite will be smeared on the friction surface, and graphite particles will even fall off, with wear debris forming between the friction surfaces. The size of graphite particles is closely related to the size of the original graphite powder, which leads to the influence of the particle size of the original graphite powder on the distribution and thickness of graphite on the friction surface.

When there is graphite between two copper micro-convex peaks, because of the lubrication of graphite, the copper micro-convex peak will not occur during cold welding, but can only occur during plastic deformation and rolling into sheet morphology.

When there are copper particles between two copper micro-convex peaks, the friction and wear behavior is similar to the normal interaction between two copper micro-convex peaks, which increases the rolling of copper particles. The particle size of the original copper powder directly affects the size of the intermediate copper particles, and then affects the surface morphology.

In the continuous service of current-carrying friction, the uniformity and thickness of graphite film between friction surfaces have an important influence on the mechanical friction and wear behavior. The graphite film is uneven, which leads to the intermittent adhesion behavior of the worn surface. With less rolling deformation of the copper micro-convex peak, there is discontinuity of the furrow morphology and the large surface roughness.

The surface quality of the friction surface also has an important influence on mechanical wear behavior. When the surface is rough, the number of actual contact micro-convex peaks is small, the stress on a single micro-convex peak is large, and thus it is easy to destroy the surface film, form cold welding tear, and further destroy the surface quality. When the surface is smooth, the stress on a single micro-convex peak is small, the graphite film is not easy to destroy, the rolling deformation of copper micro-convex peak is sufficient, and the friction surface is less destroyed.

Because the mechanical wear surface had a large amount of graphite, and appeared in a large number of rolling deformation photos, it was shown that a continuous graphite film was formed on the surface. The particle size of the copper-coated graphite was small, and the furrow deformation of wear surface was intermittent, which indicates that the graphite film on the surface was not uniform, and that there was cold welding and tearing in the friction process. The composites prepared with 150 μm copper powder and 75 μm copper coated graphite powder had the best surface quality, the fullest rolling deformation, and the best wear properties.

Arc erosion mainly includes melting and splashing [27]. After the arc is produced, a large amount of arc heat is released, and a metal molten pool is formed directly on the surface of the material. Because of the non-wetting with graphite, the copper material shrinks and converges after melting. When splashing occurs, the smaller scattered droplets cool down during flight. Because of surface tension, the larger scattered droplets form a spherical shape, and the flying process is short, forming jet-like solidified metal particles. The molten metal forms irregular solidified particles during solidification. Because the arc is formed in the process of contact micro-convex peak formation or separation, the heat is very large (the highest temperature of arc is more than 4000 K), which directly changes the material properties of the whole area, and thus the influence of the particle size of the original material on arc erosion is not obvious. At the same time, because of the shrinkage of liquid metal, a large amount of graphite on the surface of the material is exposed.

The reason why the carbon content on the wear surface was much higher than the content of 7.5 wt% in the prepared material was that after the graphite particles were removed from the material, after the deformation process, such as with the coating on the friction surface, the continuous self-generated, lubricated, conductive graphite film was formed on the friction surface. The content of C increased with the decrease of the particle size of Cu powder, which indicates that the degree of formation of lubricating film was different, and the smaller the copper particles were, the higher the C content on the worn surface was, and the more adequate the graphite lubrication film was, the more likely it was to improve the morphology of the friction surface during friction and wear. The effect of the copper-coated graphite particle size on the graphite content in the main area of mechanical wear was not obvious, which indicates that the effect of copper-coated graphite particle size on the formation of graphite film on the surface is also not obvious.

In the process of current-carrying friction, α spots were constantly formed and destroyed, and the number of α spots tended to be stable when the sliding friction was stable. The contact conductivity of friction pair increased with the increase of the number of α spots. When the number of α spots exceeds a certain number, the overall electrical conductivity is basically stable [28,29]. Because of the large number of α spots in this process, the current-carrying efficiency and current carrying stability were better in the current carrying friction process.

## 5. Conclusions

Copper-graphite composites were prepared by spark plasma sintering (SPS) with copper powder and copper-coated graphite powder. The effect of particle size of raw material powder on the current-carrying friction properties of copper-graphite composites was studied, and the following conclusions were obtained:

(1) The copper-graphite composites prepared by the SPS plasma sintering process had good surface bonding and dense materials.
(2) The friction coefficient of the composites decreased with the decrease of the particle size of the copper-coated graphite powder. The friction coefficient of the composites increased with the decrease of copper powder particle size. With the decrease of the particle size of copper-coated graphite powder, the wear rate of the composites decreased at first and then increased. With the decrease of the particle size of the copper powder, the wear rate of the composites increased significantly. The current-carrying properties of composites with different particle size ratios and QCr0.5 pairs were good and fluctuated little. The current-carrying friction properties of the composite materials prepared with 150 μm copper powder and 75 μm copper-coated graphite powder were the best.
(3) The wear surface can be divided into mechanical wear area and arc erosion area. The main area of arc erosion was less than 15% of the total area, and it was mainly distributed in the friction outlet area. The main forms of mechanical wear included furrow, rolling deformation, cold welding, tearing, among other effects, and the surface formed graphite film. The surface quality of the composite prepared by 150 micron copper powder and 75 micron copper-coated graphite powder was the best, the Sa was 3.22 μm, and the rolling deformation was the fullest, with there being no large tear pits and plough grooves. Carbon content on the worn surface was much higher than the graphite content in composites. The behavior of arc erosion was mainly melting and splashing, and the particle size of the original powder had little effect on it.

## Figures and Tables

**Table 1:** The content of different elements in the corresponding area in Figure 7/wt%.

Particle Size of Copper Powder/μm,	150			75			45		
Particle Size of Copper-Coated Graphite Powder/μm	150	75	45	150	75	45	150	75	45
Cu/wt%	69.1	67.2	66.2	65.4	65.3	65.2	58.8	58.3	58.4
C/wt%	26.9	28.1	31.1	33.0	32.1	33.1	39.2	39.5	39.6
O/wt%	4.0	4.3	2.7	1.6	2.6	1.7	2.0	2.2	2.0
